# Supplementary material for: Quantitative Measurement of Spinal Cerebrospinal Fluid by Cascade Artificial Intelligence Models in Patients with Spontaneous Intracranial Hypotension
Source: Biomedicines. 2022 Aug 22;10(8):2049. doi: 10.3390/biomedicines10082049 (PMC9405775; doi:10.3390/biomedicines10082049)
Supplement: Supplementary file 1 [file biomedicines-10-02049-s001.zip › biomedicines-1831317-supplementary.pdf]

Table S1. Demographics of patients and healthy volunteers.

	Total (*n* = 25)	Patients (*n* = 13, 52%)	HVs (*n* = 12, 48%)	*p* value
Age at MRI examination [a]		43.31 ± 9.83	37.17 ± 7.77	0.098
Sex[b]				
Female	8	6	2	0.202
Male	17	7	10	

Mann-Whitney test [a]. Fisher's exact test [b]. Numeric data are presented as mean ± standard deviation. HVs healthy volunteers.
